# 2-(2*H*-Tetra­zol-5-yl)pyridinium perchlorate monohydrate

**DOI:** 10.1107/S160053680905199X

**Published:** 2009-12-09

**Authors:** Jing Dai, Wen-Ni Zheng

**Affiliations:** a Ordered Matter Science Research Center, College of Chemistry and Chemical Engineering, Southeast UniVersity, Nanjing 210096, People’s Republic of China

## Abstract

In the cation of the title compound, C_6_H_6_N_5_^+^·ClO_4_^−^·H_2_O, the pyridinium and tetra­zole rings are essentially coplanar, making a dihedral angle of 1.2 (2)°. In the crystal, inter­molecular N—H⋯O and O—H⋯O hydrogen bonds link the cations, anions and water mol­ecules into a ribbon-like structure along the *c* axis. Adjacent ribbons are linked via π–π stacking inter­actions between the tetra­zole rings, with a centroid–centroid distance of 3.484 (2) Å.

## Related literature

For applications of tetra­zole derivatives in coordination chemistry, see: Zhao *et al.* (2008 ▶); Fu *et al.* (2008 ▶, 2009 ▶). For related structures, see: Fu *et al.* (2007 ▶); Fu & Xiong (2008 ▶).
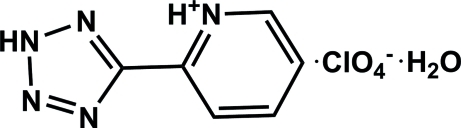


## Experimental

### 

#### Crystal data

C_6_H_6_N_5_^+^·ClO_4_^−^·H_2_O
*M* *_r_* = 265.62
Triclinic, 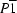

*a* = 7.9945 (16) Å
*b* = 8.8679 (18) Å
*c* = 9.4184 (19) Å
α = 78.28 (3)°
β = 70.20 (3)°
γ = 67.97 (3)°
*V* = 580.1 (2) Å^3^
*Z* = 2
Mo *K*α radiation
μ = 0.35 mm^−1^
*T* = 298 K
0.40 × 0.35 × 0.20 mm

#### Data collection

Rigaku Mercury2 diffractometer
Absorption correction: multi-scan (*CrystalClear*; Rigaku, 2005 ▶) *T*_min_ = 0.881, *T*_max_ = 0.940
6033 measured reflections
2661 independent reflections
2081 reflections with *I* > 2σ(*I*)
*R*_int_ = 0.026

#### Refinement

*R*[*F* ^2^ > 2σ(*F* ^2^)] = 0.065
*wR*(*F* ^2^) = 0.195
*S* = 1.04
2661 reflections
154 parameters
H-atom parameters constrained
Δρ_max_ = 0.53 e Å^−3^
Δρ_min_ = −0.61 e Å^−3^

### 

Data collection: *CrystalClear* (Rigaku, 2005 ▶); cell refinement: *CrystalClear*; data reduction: *CrystalClear*; program(s) used to solve structure: *SHELXS97* (Sheldrick, 2008 ▶); program(s) used to refine structure: *SHELXL97* (Sheldrick, 2008 ▶); molecular graphics: *SHELXTL* (Sheldrick, 2008 ▶); software used to prepare material for publication: *SHELXTL*.

## Supplementary Material

Crystal structure: contains datablocks I, New_Global_Publ_Block. DOI: 10.1107/S160053680905199X/ci2980sup1.cif

Structure factors: contains datablocks I. DOI: 10.1107/S160053680905199X/ci2980Isup2.hkl

Additional supplementary materials:  crystallographic information; 3D view; checkCIF report

## Figures and Tables

**Table 1:** Hydrogen-bond geometry (Å, °)

*D*—H⋯*A*	*D*—H	H⋯*A*	*D*⋯*A*	*D*—H⋯*A*
N3—H3⋯O4	0.90	1.76	2.640 (4)	166
N1—H1⋯O1*W*^i^	0.86	1.79	2.633 (4)	166
O1*W*—H1*WB*⋯O3	0.72	2.06	2.778 (4)	172
O1*W*—H1*WA*⋯O2^ii^	0.78	1.99	2.771 (4)	174

Symmetry codes: (i) 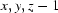; (ii) 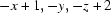.

**Fig. 1.**
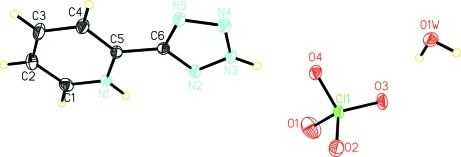
The asymmetric unit of the title compound with the atomic numbering scheme. Displacement ellipsoids are drawn at the 30% probability level.

**Fig. 2.**
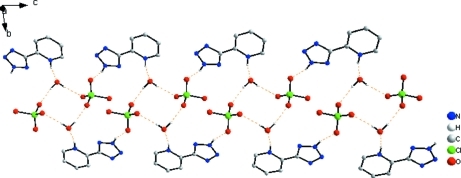
The crystal packing of the title compound, viewed along the a axis, showing the two dimensionnal hydrogen-bonded network. H atoms not involved in hydrogen bonding (dashed line) have been omitted for clarity.

## supplementary crystallographic information

### Comment

In the past few years, more and more people have focused on the chemistry of tetrazole derivatives because of their multiple coordination modes as ligands to metal ions and for the construction of novel metal-organic frameworks (Zhao *et al.*, 2008; Fu *et al.*, 2008). As an extension of these work on the structure and properties (Fu *et al.*, 2007; Fu & Xiong 2008), we report here the crystal structure of the title compound 2-(2*H*-tetrazol-5-yl)pyridinium perchlorate monohydrate.

In the title compound (Fig.1), the pyridine N atom is protonated. The pyridinium and tetrazole rings are essentially coplanar, with the dihedral angle between them being 1.2 (2)°. The geometric parameters of the tetrazole rings are comparable to those in related structures (Zhao *et al.*, 2008; Fu *et al.*, 2009).

The crystal packing is stabilized by N—H···O and O—H···O hydrogen bonds. These hydrogen bonds link the ionic units and water molecules to form a ribbon like structure parallel to the *c* axis (Table 1 and Fig.2).

### Experimental

Picolinonitrile (30 mmol), NaN_3_ (45 mmol), NH_4_Cl (33 mmol) and DMF (50 ml) were added in a flask under nitrogen atmosphere and the mixture was stirred at 110°C for 20 h. The resulting solution was then poured into ice-water (100 ml), and a white solid was obtained after adding HCl (6 *M*) till pH = 6. The precipitate was filtered and washed with distilled water. Colourless block-shaped crystals suitable for X-ray analysis were obtained from the crude product by slow evaporation of a water-HClO_4_ (50:1 *v*/*v*) solution.

### Refinement

All H atoms attached to C and N atoms were fixed geometrically and treated as riding with C–H = 0.93 Å (aromatic), N–H = 0.86 Å (pyridine N) and N–H = 0.90 Å (tetrazole N) with *U*_iso_(H) =1.2U_eq_(C,N). H atoms of the water molecule were located in difference Fourier maps and freely refined. In the last stage of the refinement they were treated as riding on the O atom, with *U*_ĩso_(H) = 1.5*U_eq_*(O).

### Crystal data

**Table:**

C_6_H_6_N_5_^+^·ClO_4_^−^·H_2_O	*Z* = 2
*M**_r_* = 265.62	*F*(000) = 272
Triclinic, *P*1	*D*_x_ = 1.521 Mg m^−^^3^
Hall symbol: -P 1	Mo *K*α radiation, λ = 0.71073 Å
*a* = 7.9945 (16) Å	Cell parameters from 2081 reflections
*b* = 8.8679 (18) Å	θ = 3.1–27.5°
*c* = 9.4184 (19) Å	µ = 0.35 mm^−^^1^
α = 78.28 (3)°	*T* = 298 K
β = 70.20 (3)°	Block, colourless
γ = 67.97 (3)°	0.40 × 0.35 × 0.20 mm
*V* = 580.1 (2) Å^3^	

### Data collection

**Table:**

Rigaku Mercury2 diffractometer	2661 independent reflections
Radiation source: fine-focus sealed tube	2081 reflections with *I* > 2σ(*I*)
graphite	*R*_int_ = 0.026
Detector resolution: 13.6612 pixels mm^-1^	θ_max_ = 27.5°, θ_min_ = 3.1°
ω scans	*h* = −10→10
Absorption correction: multi-scan (*CrystalClear*; Rigaku, 2005)	*k* = −11→11
*T*_min_ = 0.881, *T*_max_ = 0.940	*l* = −12→12
6033 measured reflections	

### Refinement

**Table:**

Refinement on *F*^2^	Primary atom site location: structure-invariant direct methods
Least-squares matrix: full	Secondary atom site location: difference Fourier map
*R*[*F*^2^ > 2σ(*F*^2^)] = 0.065	Hydrogen site location: inferred from neighbouring sites
*wR*(*F*^2^) = 0.195	H-atom parameters constrained
*S* = 1.04	*w* = 1/[σ^2^(*F*_o_^2^) + (0.0988*P*)^2^ + 0.7631*P*] where *P* = (*F*_o_^2^ + 2*F*_c_^2^)/3
2661 reflections	(Δ/σ)_max_ = 0.001
154 parameters	Δρ_max_ = 0.53 e Å^−^^3^
0 restraints	Δρ_min_ = −0.61 e Å^−^^3^

### Special details

**Table:**

Geometry. All e.s.d.'s (except the e.s.d. in the dihedral angle between two l.s. planes) are estimated using the full covariance matrix. The cell e.s.d.'s are taken into account individually in the estimation of e.s.d.'s in distances, angles and torsion angles; correlations between e.s.d.'s in cell parameters are only used when they are defined by crystal symmetry. An approximate (isotropic) treatment of cell e.s.d.'s is used for estimating e.s.d.'s involving l.s. planes.
Refinement. Refinement of *F*^2^ against ALL reflections. The weighted *R*-factor *wR* and goodness of fit *S* are based on *F*^2^, conventional *R*-factors *R* are based on *F*, with *F* set to zero for negative *F*^2^. The threshold expression of *F*^2^ > σ(*F*^2^) is used only for calculating *R*-factors(gt) *etc*. and is not relevant to the choice of reflections for refinement. *R*-factors based on *F*^2^ are statistically about twice as large as those based on *F*, and *R*- factors based on ALL data will be even larger.

### Fractional atomic coordinates and isotropic or equivalent isotropic displacement parameters (Å^2^)

**Table:**

	*x*	*y*	*z*	*U*_iso_*/*U*_eq_	
N1	0.2411 (4)	0.5225 (3)	0.0449 (3)	0.0337 (6)	
H1	0.3305	0.4346	0.0590	0.040*	
N2	0.3244 (4)	0.4372 (3)	0.3287 (3)	0.0398 (7)	
N3	0.3065 (4)	0.4464 (3)	0.4707 (3)	0.0411 (7)	
H3	0.3806	0.3754	0.5256	0.049*	
N4	0.1668 (5)	0.5728 (4)	0.5322 (3)	0.0459 (7)	
N5	0.0848 (4)	0.6535 (3)	0.4264 (3)	0.0412 (7)	
C1	0.2097 (6)	0.5603 (5)	−0.0901 (4)	0.0445 (8)	
H1A	0.2857	0.4927	−0.1676	0.053*	
C2	0.0668 (6)	0.6976 (5)	−0.1161 (4)	0.0525 (10)	
H2A	0.0448	0.7239	−0.2104	0.063*	
C3	−0.0443 (6)	0.7964 (5)	0.0006 (4)	0.0517 (10)	
H3A	−0.1431	0.8896	−0.0144	0.062*	
C4	−0.0087 (5)	0.7568 (4)	0.1392 (4)	0.0442 (8)	
H4A	−0.0825	0.8233	0.2178	0.053*	
C5	0.1372 (4)	0.6176 (4)	0.1602 (3)	0.0330 (7)	
C6	0.1842 (4)	0.5680 (4)	0.3032 (3)	0.0323 (7)	
Cl1	0.58684 (12)	0.09352 (10)	0.68911 (9)	0.0393 (3)	
O1	0.7478 (6)	0.0326 (6)	0.5118 (5)	0.0995 (13)	
O2	0.4562 (4)	0.0041 (4)	0.7313 (3)	0.0565 (7)	
O3	0.6963 (4)	0.0591 (3)	0.7938 (3)	0.0557 (8)	
O4	0.4923 (4)	0.2690 (3)	0.6656 (3)	0.0501 (7)	
O1W	0.5256 (4)	0.2475 (3)	1.0393 (3)	0.0483 (7)	
H1WA	0.5261	0.1815	1.1080	0.073*	
H1WB	0.5728	0.2054	0.9710	0.073*	

### Atomic displacement parameters (Å^2^)

**Table:**

	*U*^11^	*U*^22^	*U*^33^	*U*^12^	*U*^13^	*U*^23^
N1	0.0345 (14)	0.0324 (13)	0.0324 (13)	−0.0090 (11)	−0.0084 (11)	−0.0057 (10)
N2	0.0450 (16)	0.0349 (14)	0.0343 (14)	−0.0017 (12)	−0.0165 (12)	−0.0060 (11)
N3	0.0482 (16)	0.0363 (15)	0.0360 (14)	−0.0055 (13)	−0.0197 (13)	−0.0010 (11)
N4	0.0537 (18)	0.0441 (16)	0.0345 (15)	−0.0054 (14)	−0.0158 (13)	−0.0079 (12)
N5	0.0465 (16)	0.0368 (15)	0.0326 (14)	−0.0002 (12)	−0.0140 (12)	−0.0088 (11)
C1	0.055 (2)	0.049 (2)	0.0323 (17)	−0.0221 (18)	−0.0079 (15)	−0.0086 (14)
C2	0.062 (2)	0.062 (2)	0.0369 (18)	−0.019 (2)	−0.0240 (17)	0.0028 (17)
C3	0.051 (2)	0.049 (2)	0.048 (2)	−0.0013 (17)	−0.0260 (18)	0.0017 (17)
C4	0.0438 (19)	0.0415 (19)	0.0392 (18)	−0.0013 (15)	−0.0151 (15)	−0.0058 (14)
C5	0.0333 (15)	0.0332 (15)	0.0315 (15)	−0.0083 (13)	−0.0110 (12)	−0.0030 (12)
C6	0.0336 (15)	0.0296 (15)	0.0309 (15)	−0.0059 (12)	−0.0094 (12)	−0.0053 (12)
Cl1	0.0429 (5)	0.0358 (4)	0.0364 (4)	−0.0004 (3)	−0.0210 (3)	−0.0054 (3)
O1	0.086 (3)	0.122 (3)	0.083 (3)	−0.016 (3)	−0.014 (2)	−0.046 (2)
O2	0.0581 (17)	0.0598 (17)	0.0539 (16)	−0.0220 (14)	−0.0222 (13)	0.0051 (13)
O3	0.0647 (17)	0.0527 (16)	0.0510 (15)	0.0011 (13)	−0.0408 (14)	−0.0056 (12)
O4	0.0583 (16)	0.0351 (13)	0.0477 (14)	0.0082 (11)	−0.0281 (12)	−0.0083 (11)
O1W	0.0575 (16)	0.0363 (13)	0.0405 (13)	−0.0069 (12)	−0.0088 (11)	−0.0067 (10)

### Geometric parameters (Å, °)

**Table:**

N1—C1	1.331 (4)	C2—H2A	0.93
N1—C5	1.348 (4)	C3—C4	1.378 (5)
N1—H1	0.86	C3—H3A	0.93
N2—N3	1.312 (4)	C4—C5	1.378 (5)
N2—C6	1.324 (4)	C4—H4A	0.93
N3—N4	1.314 (4)	C5—C6	1.458 (4)
N3—H3	0.90	Cl1—O2	1.446 (3)
N4—N5	1.318 (4)	Cl1—O3	1.447 (3)
N5—C6	1.354 (4)	Cl1—O4	1.460 (3)
C1—C2	1.368 (6)	Cl1—O1	1.768 (4)
C1—H1A	0.93	O1W—H1WA	0.78
C2—C3	1.382 (6)	O1W—H1WB	0.72
			
C1—N1—C5	122.0 (3)	C2—C3—H3A	120.0
C1—N1—H1	119.0	C3—C4—C5	119.4 (3)
C5—N1—H1	119.0	C3—C4—H4A	120.3
N3—N2—C6	101.6 (3)	C5—C4—H4A	120.3
N2—N3—N4	114.4 (3)	N1—C5—C4	119.2 (3)
N2—N3—H3	125.6	N1—C5—C6	118.2 (3)
N4—N3—H3	120.0	C4—C5—C6	122.6 (3)
N3—N4—N5	106.4 (3)	N2—C6—N5	112.5 (3)
N4—N5—C6	105.1 (3)	N2—C6—C5	125.1 (3)
N1—C1—C2	120.7 (3)	N5—C6—C5	122.3 (3)
N1—C1—H1A	119.7	O2—Cl1—O3	113.55 (17)
C2—C1—H1A	119.7	O2—Cl1—O4	111.66 (17)
C1—C2—C3	118.7 (3)	O3—Cl1—O4	110.80 (16)
C1—C2—H2A	120.7	O2—Cl1—O1	107.3 (2)
C3—C2—H2A	120.7	O3—Cl1—O1	107.05 (19)
C4—C3—C2	120.0 (4)	O4—Cl1—O1	106.0 (2)
C4—C3—H3A	120.0	H1WA—O1W—H1WB	107.6

### Hydrogen-bond geometry (Å, °)

**Table:**

*D*—H···*A*	*D*—H	H···*A*	*D*···*A*	*D*—H···*A*
N3—H3···O4	0.90	1.76	2.640 (4)	166
N1—H1···O1W^i^	0.86	1.79	2.633 (4)	166
O1W—H1WB···O3	0.72	2.06	2.778 (4)	172
O1W—H1WA···O2^ii^	0.78	1.99	2.771 (4)	174

Symmetry codes: (i) *x*, *y*, *z*−1; (ii) −*x*+1, −*y*, −*z*+2.
